# Systematic mining and engineering of signal peptides to achieve efficient secretion of cephalosporin C acylase in *Escherichia coli*

**DOI:** 10.1016/j.synbio.2026.03.006

**Published:** 2026-03-24

**Authors:** Xinyi Ren, Zhiying Yang, Siyu Chen, Chunxiang Pu, Huiying Wang, Jinlong Li, Qichen Cao, Xuyang Huang, Gang Fu, Bo Yuan, Jianmei Luo, Zhoutong Sun, Huina Dong, Dawei Zhang

**Affiliations:** a Tianjin University of Science and Technology, Tianjin, China; b Tianjin Institute of Industrial Biotechnology, Chinese Academy of Sciences, Tianjin, China; c University of Chinese Academy of Sciences, Beijing, China; d State Key Laboratory of Engineering Biology for Low-Carbon Manufacturing, Tianjin Institute of Industrial Biotechnology, Chinese Academy of Sciences, Tianjin, China

**Keywords:** Cephalosporin C acylase, Signal peptide mining, Signal peptide engineering, Protein secretion, *Escherichia coli*

## Abstract

Cephalosporin C acylases (CCAs) catalyze the hydrolysis of cephalosporin C to 7-aminocephalosporanic acid, a key intermediate for semisynthetic cephalosporin antibiotics. The functional secretion of heterologous CCAs in *Escherichia coli* is often constrained by signal peptide efficiency. To enhance the production of the engineered CCA mutant A14 from *Bosea* sp. OK403, we performed systematic signal peptide screening and identified the native SP_AsPGA_ as most effective. Following codon optimization to generate SP_AsPGA∗_, targeted mutagenesis of its N-, H-, and C-regions produced the superior H9 mutant (C16A). This variant increased extracellular A14 expression and activity by 6.26-fold and 2.0-fold, respectively. Molecular dynamics simulations indicated the C16A substitution stabilizes the signal peptide conformation and facilitates SecA translocon interaction. This work establishes that systematic mining and engineering of signal peptides, particularly through mutations that enhance structural stability, is a powerful strategy for optimizing the secretory production of complex enzymes in recombinant systems.

## Graphical abstract

**Figure:**
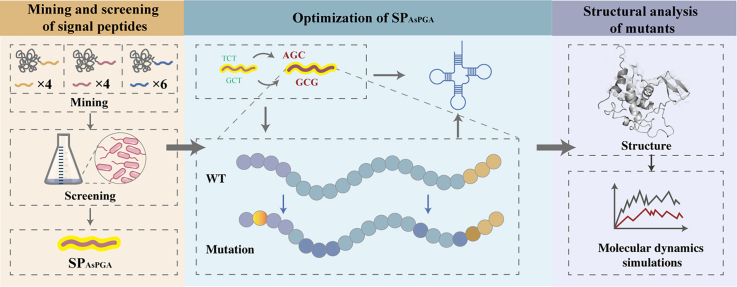
Strategy to enhance CPC acylase secretion in *Escherichia coli* via signal peptide mining, screening, and optimization, followed by structural analysis and molecular dynamics simulations of the resulting mutants.

## 1 Introduction

β-lactam antibiotics belong to a class of antibacterial drugs, and they account for 65% of the global antibiotic market [1]. Among them, cephalosporins are one of the most widely produced and consumed antibiotics globally, accounting for approximately 30 % of all antibiotic consumption [2]. 7-Amino cephalosporanic acid (7-ACA) is one of the core nuclei for synthesizing β-lactam antibiotics. It is derived from cephalosporin C (CPC), which was originally isolated from the fungus *Cephalosporium acremonium* [3]. CPC is converted to 7-ACA by either a chemical or an enzyme derived catalytic removal of the 7-amino adipoyl side chain [4].

Enzymatic transformation of CPC to 7-ACA is the best alternative and has industrial significance [5]. Previously, the industrial production of 7-ACA from CPC predominantly involved a two-step enzymatic process. The initial step employed d-amino acid oxidase (DAAO) to oxidize CPC into α-ketoadipyl-7-ACA, which was subsequently converted to glutaryl-7-ACA (GL-7-ACA) and then hydrolyzed to yield 7-ACA [6]. In contrast, one-step enzymatic conversion catalyzed by Cephalosporin C acylase (CCA) offers a streamlined and economically advantageous alternative by directly transforming CPC into 7-ACA, thereby eliminating the production of harmful hydrogen peroxide associated with the oxidative step [7].

The gene structure encoding CCAs exhibits variation among different enzymes, however, it generally comprises a signal peptide followed by the α-subunit, a spacer sequence, and subsequently the β-subunit [8]. For proteins destined for export to the periplasm, translocation across the host membrane severely limits their production. Specifically, during protein overexpression, the translocation process becomes saturated, which leading to a substantial decline in efficiency [9]. Signal peptides play a critical role in mediating the translocation of proteins across biological membranes [10]. The CCA derived from *Bosea* sp. OK403, which was characterized in our previous work, does not contain signal peptide (Fig. S1 in Supplementary material 1) [7]. Creating or modifying a suitable signal peptide for it may be helpful in enhancing its expression, especially in increasing its extracellular expression level.

**Fig. 1:**
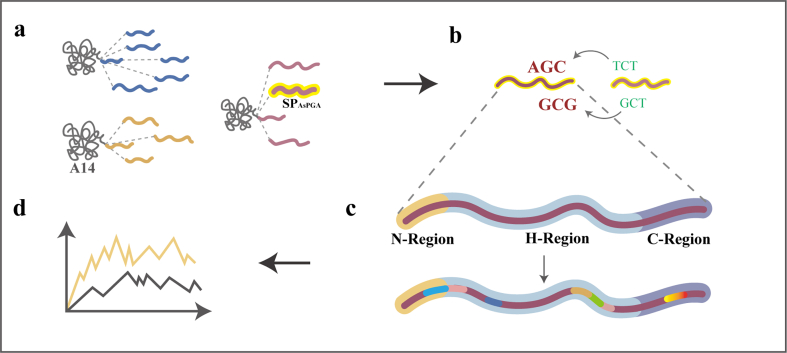
The flow diagram of experimental strategies in this study. (a) Mining of signal peptides to enhance the secretion of protein A14. (b–c) Site-directed mutagenesis of SP_AsPGA_ to enhance the secretion of protein A14. (d) C16A stabilizes signal peptide conformation to promote protein translocation via SecA.

In this study, we attempted to increase the expression and secretion of the CCA mutant A14 derived from *Bosea* sp. OK403 in *Escherichia coli* BL21(DE3). Firstly, the signal peptide was identified through a mining and screening process adapted to the host system (Fig. 1a). Furthermore, the coding sequence of the most efficient signal peptide was codon-optimized, and site-directed mutagenesis was performed within its N-, H- and C-regions (Fig. 1b and c). Finally, molecular dynamics simulations to compare the conformational changes of the mutants and wild-type protein were performed, aiming to clarify their functional differences (Fig. 1d).

## 2 Materials and methods

### 2.1 Strains, plasmids, and cultivation conditions

Plasmids and bacteria strains used in this study were listed in Table S1 and S2, respectively. Luria-Bertani (LB) medium contained 10 g/L Bacto peptone, 5 g/L yeast extract and 10 g/L NaCl. TB medium contained 12 g/L tryptone, 24 g/L yeast extract, 4 mL/L glycerol, 12.54 g/L K_2_HPO_4_ and 2.31 g/L KH_2_PO_4_. The *E. coli* strains were inoculated in 5 mL of LB medium overnight at 37 °C at a stirring speed of 220 rpm. 300 μL of these culture were inoculated to 30 mL of TB medium containing 50 μg/mL kanamycin in 250-mL Erlenmeyer flasks and then incubated at 37 °C until an optical density at 600 nm (OD_600_) reached 0.6-0.8. Subsequently, 0.8 mM isopropyl thio-β-d-galactopyranoside (IPTG) was added to induce protein expression at 25 °C for 20 h. Cell growth was monitored by measurement of the optical density at 600 nm (OD_600_).

### 2.2 DNA manipulation and plasmid construction

All the primers used in this study are listed in Table S3. Plasmids were constructed using the megaprimer method and PrimeSTAR Max DNA polymerase from Takara. PCR products using pET28a-A14 served as the template for fragment amplification were analyzed by electrophoresis on an agarose gel. 0.5 μL of *Dpn*I was added to 10 μL of PCR reaction mix and digested at 37 °C for 1 h. After *Dpn*I digestion, PCR products were transformed into electrocompetent *E. coli* BL21(DE3) cells to generate a mutant strain and the introduced mutations were confirmed by Sanger sequencing for rapid quality control and screening (GENEWIZ Company). Green Taq Mix was used to do the colonies PCR (Vazyme Biotech).

### 2.3 SDS-PAGE analysis

The cells were harvested via centrifugation at 6000 rpm for 3 min at 4 °C, collected the supernatant fraction and re-suspended the pellet in ddH_2_O. Then, the cell suspension was disrupted by sonication on ice bath and separated by centrifugation at 12,000 rpm for 2 min at 4 °C. The supernatant and the culture medium supernatant containing soluble proteins were assayed for CCAs expression. The pellet containing insoluble proteins and cell debris was washed with ddH_2_O. The expression results of the CCAs were measured using sodium dodecyl sulfate polyacrylamide gel electrophoresis (SDS-PAGE) with 4–20% separating gel (SurePAGE™). Rainbow 180 Protein Marker (Solarbio, Beijing, China) was used to determine the apparent molecular weight of separated proteins. Proteins were visualized with Coomassie Brilliant Blue. Measurements of soluble target protein, inclusion bodies, and total proteins were performed by grey scale scanning method using ImageJ (version 1.53t; National Institutes of Health, Bethesda, MD, USA) software.

### 2.4 Proteomic identification

The target band (35–48 kDa) of SDS-PAGE gel was sliced and cut into pieces, the protein was in-gel digested followed the description of reference [11]. Briefly, the gel pieces were destained with 100 mM ammonium bicarbonate/acetonitrile (1:1, *v/v*), and then were reduced and alkylated with 10 mM Dithiothreitol (DTT) and 40 mM Iodoacetoamide (IAM) solution respectively. The gel pieces were shrunken with neat acetonitrile and then trypsin buffer (50 mM ammonium bicarbonate) was added. The gel was incubated under 37 °C overnight. The digested peptides were extracted with acetonitrile and were dried under vacuum. The resulting peptides were dissolved with 0.1% formic acid and were injected into the LC-MS system for analysis.

The peptides were analyzed by Thermo Orbitrap Eclipse mass spectrometry coupled with Thermo vanquish neo nano-LC system. A homemade 75 μm × 25 cm capillary column (ReprosilPur 120 C18-ÀQ, 1.9 μm, Dr. Maisch) was used. The flow rate was set as 300 nL/min. Mobile phase A was 0.1% formic acid and mobile phase B was 0.1% fomic acid/acetonitrile (2:8, v/v). The peptides were separated with the gradient of mobile phase B from 5–12% for 2 min, 12–30% for 18 min, 30–45% for 3.5 min, 45–95% for 3 min, and maintained 100% for 3.5 min. The column oven temperature was set as 55 °C. The mass spectrometer was operated under DDA (data dependent acquisition) mode. The spray voltage of ESI source was set to 2000 V. The MS1 scan range was *m*/*z* 300–500 with the resolution of 60000. The MS2 collision type was set as HCD, the NCE was set to 32 and the maxIT was set to 50 ms. The MS2 scan range was set as “auto”. The total cycle time was set to 1.3 s.

The obtained RAW file was searched against the Uniprot *E. coli* K12 fasta file (6421 entries) using Proteome Discoverer (*v3.0*, Thermo). Carbamidomethyl (C) was set as static modification, Acetyl (Protein N-term), Met-loss (M) (Protein N-term) and Met-loss + Acetyl (M) (Protein N-term) were set as the variable modification. At most 2 miss cleavages were allowed. A mass error of ±10 ppm was set to MS1 and mass error of ±0.02 Da was set to MS2. The protein level FDR (False Discovery Rate) was set to 1%。

Candidate signal peptides were extracted from the N-terminal regions of the identified proteins. Only proteins containing a predicted N-terminal signal peptide were considered, based on Signal P prediction. Signal peptides were categorized as Sec- or Tat-type according to the presence of the twin-arginine motif. The selected signal peptide sequences were then fused to the N-terminus of A14, replacing the original signal peptide, and the resulting constructs were evaluated for extracellular enzyme activity and supernatant protein accumulation.

### 2.5 Assay of CCAs activity

An aliquot (500 μL) of the bacterial culture was centrifuged (5000 rpm, 10 min, RT). The supernatant was collected and stored at 4 °C. The cell pellet was subjected to freeze-thaw cycling (−80 °C, 60 min; RT, 30 min) and then resuspended in 200 μL of 0.1 M potassium phosphate buffer (pH 8.0) containing 1 mg/mL lysozyme. The suspension was incubated sequentially at 37 °C for 60 min and 45 °C for 60 min. After centrifugation (5000 rpm, 20 min, 4 °C), the clarified lysate (20 μL) was mixed with 200 μL of substrate reaction solution (0.1 M potassium phosphate buffer, pH 8.0, containing 2% w/v CPC) and incubated at 37 °C for 12 h. The reaction was terminated by adding 200 μL of a termination solution (0.05 M NaOH: 20% glacial acetic acid = 1:2, v/v). The mixture was centrifuged (5000 rpm, 10 min), and 200 μL of the resulting supernatant was transferred to a 96-well plate. Then, 40 μL of color-developing solution (0.5% w/v *p*-Dimethylaminobenzaldehyde in methanol) was added in the dark. After 10 min of incubation at RT, the absorbance was measured at 415 nm. Enzyme activity (U) was defined as the amount of enzyme required to hydrolyze 1 μmol of CPC per minute at 37 °C. All experiments were performed in triplicate, and data are presented as mean ± standard deviation.

### 2.6 Molecule dynamic simulations

Structural prediction and modeling of the signal peptide-SecA complex was performed using AlphaFold3 [12]. Following preparation, the 3D models of SP_AsPGA_-SecA, H9-SecA and H10-SecA were employed as the initial complex for molecular dynamics (MD) simulations. Initially, the constructed models were prepared and subjected to energy minimization using the AMBER22 [13] and the ff14SB/GAFF2 force fields. For ligand molecules, force field parameters were derived using the ANTECHAMBER module and GAFF2 force field, combined with AM1-BCC charges [14]. During minimization, the system underwent a stepwise equilibration protocol. And then, it was first heated to 310 K over 100 ps under constant volume conditions, with positional restraints of 20 kcal·mol^−1^ applied to the protein backbone atoms (@CA, C, O, N). This was followed by a 50 ps unrestrained equilibration at constant pressure (NPT). Subsequently, 100 ns NPT production simulations were performed at 310 K and 1 atm pressure with a 2-fs integration time step. The simulation system were solvates in a TIP3P water box with periodic boundary conditions applied [15]. Long-range electrostatic were computer using the Particle Mesh Ewald (PME) method integrated with the AMBER package. All the bonds involving hydrogen were restrained using the SHAKE algorithm throughout the simulations [16]. Temperature and pressure were controlled using a Langevin thermostat and an anisotropic Monte-Carlo barostat [17], respectively. All simulations were accelerated using the GPU PMEMD module implemented on NVIDIA GeForce 30 Series cards [18]. Equilibrium MD simulation trajectories were analyzed using the CPPTRAJ [19] module of AMBERTOOLs.

## 3 Results and discussion

### 3.1 Mining of signal peptides to enhance the secretion of protein A14

**Fig. 2:**
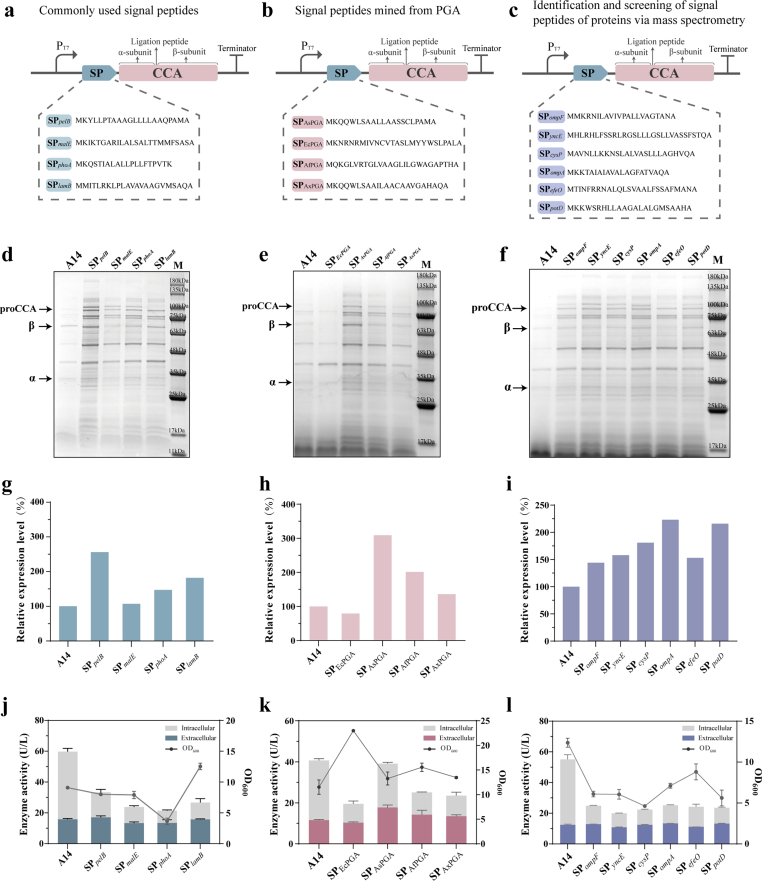
Systematic evaluation of signal peptides for enhanced secretion of A14. (a–c) Schematic diagrams of the expression plasmids, featuring A14 fused to: (a) common signal peptides, (b) signal peptides derived from PGAs, and (c) signal peptides identified from highly secreted host proteins via mass spectrometry. (d–f) SDS-PAGE analysis of A14 expression in the culture supernatant of strains harboring the corresponding plasmids. (g–i) Densitometric quantification of extracellular A14 levels from the SDS-PAGE gels (d–f). (j–l) Measurement of intracellular and extracellular A14 enzyme activity for strains containing the corresponding plasmids.

In order to enhance the secretion of A14, it is necessary to screen for efficient signal peptides that function well in *E. coli*. The signal peptides from three different strategies were added at the N-terminus of A14 to facilitate its proper folding and secretion, including commonly used signal peptides, signal peptides of penicillin G acylases (PGAs) from different bacterial strains, and signal peptides of proteins screened and identified via mass spectrometry (Fig. 2, Table 1). This comprehensive design was predicated on the hypothesis that these sequences would direct the nascent A14 polypeptide to the secretion machinery, thereby facilitating its correct folding and subsequent translocation across the cytoplasmic membrane. The rationale for this strategy was twofold. First, it leveraged the known efficacy of established signal peptides while probing for potential specialization by testing those natively associated with PGA enzymes, which share a functional context with A14. Second, the inclusion of mass spectrometry-identified signals aimed to uncover novel, high-performance sequences that might be uniquely suited for this specific expression host and target protein. This multi-faceted screening platform allowed us to empirically determine the most effective signal peptide for A14 secretion, moving beyond a trial-and-error approach towards a more rational and informed selection process.

**Table 1:** The signal peptides used in this study.

No.	SP	Sequences	Length (aa)	Type	Origins	GRAVY value
Commonly used signal peptides						

1	SP_*lamB*_	MMITLRKLPLAVAVAAGVMSAQA	23	Sec	*E.coli*	1.287
2	SP_*pelB*_	MKYLLPTAAAGLLLLAAQPAMA	22	Sec	*E.coli*	1.191
3	SP_*phoA*_	MKQSTIALALLPLLFTPVTK	20	Sec	*E.coli*	0.930
4	SP_*malE*_	MKIKTGARILALSALTTMMFSASA	24	Sec	*E.coli*	0.863

Signal peptides mined from PGAs						

1	SP_AsPGA_	MKQQWLSAALLAASSCLPAMA	21	Sec	*Achromobacter* sp. CCM 4824	0.786
2	SP_EcPGA_	MKNRNRMIVNCVTASLMYYWSLPALA	26	Sec	*E.coli*	0.273
3	SP_AfPGA_	MQKGLVRTGLVAAGLILGWAGAPTHA	26	Sec	*Alcaligenes faecalis* strain PX16	0.692
4	SP_AxPGA_	MKQQWLSAAILAACAAVGAHAQA	23	Sec	*Achromobacter xylosoxidans* strain PX02	0.748

Proteomic identification of screened signal peptides						
SP1	SP_*ompF*_	MMKRNILAVIVPALLVAGTANA	22	Sec	*E.coli*	1.259
SP2	SP_*yncE*_	MHLRHLFSSRLRGSLLLGSLLVASSFSTQA	30	Tat	*E.coli*	0.507
SP3	SP_*cysP*_	MAVNLLKKNSLALVASLLLAGHVQA	25	Sec	*E.coli*	1.064
SP4	SP_*ompA*_	MKKTAIAIAVALAGFATVAQA	21	Sec	*E.coli*	1.295
SP5	SP_*efeO*_	MTINFRRNALQLSVAALFSSAFMANA	26	Tat	*E.coli*	0.654
SP6	SP_*potD*_	MKKWSRHLLAAGALALGMSAAHA	23	Sec	*E.coli*	0.417

Firstly, four commonly used signal peptides, including SP_*lamB*_, SP_*pelB*_, SP_*phoA*_, and SP_*malE*_, which had been widely used for efficient secretory production of recombinant proteins in *E. coli* [20,21], were selected to add to the N-terminus of A14. Compared to A14, these four recombinant strains all exihibited increased expression of CCAs, and the SP_*pelB*_ yielded the most effective outcome (Fig. 2a), which indicates that the addition of the signal peptide have a positive effect on CCAs extracellular expression. To precisely compare the differences between these four recombinant strains, the relative expression levels of CCAs were estimated by densitometry analysis of the electrophoresis bands via the ImageJ Software. The SP_*pelB*_ strain demonstrated a significantly higher relative expression level, which was 2.5-fold that of the control A14 (Fig. 2d). This enhanced expression correlated with the highest extracellular enzyme activity, recorded at 1.1-fold that of the control (Fig. 2g). They were both significantly greater than those of the other engineered strains.

In addition, given the structural homology between PGA and CCA, we hypothesized that fusing the native PGA signal peptide to the N-terminus of A14 would enhance its extracellular secretion. Thus, the signal peptides of PGAs from *E.* *coli* (SP_EcPGA_), *Achromobacter* sp. CCM 4824 (SP_AsPGA_), *Alcaligenes faecalis* (SP_AfPGA_), and *Achromobacter xylosoxidans* (SP_AxPGA_) were selected to add to the N-terminus of A14, respectively. The signal peptide sequences of PGAs were examined using the SignalP server (version 6). The SP_EcPGA_ strain exhibited reduced extracellular protein secretion of SP_EcPGA_-A14 fusion compared to the control A14, while the recombinant strains contained SP_AsPGA_, SP_AfPGA_ and SP_AxPGA_ all demonstrated higher secretory expression levels of CCA (Fig. 2b). Not only did the SP_AsPGA_ strain exihibit a 3-fold increase in relative expression level over the A14 control (Fig. 2e), but it also achieved a 1.5-fold higher extracellular enzyme activity (Fig. 2h), both of which were significantly greater than those of the other engineered strains.

Furthermore, during the initial secretion screening experiments, we consistently observed a prominent protein band in the culture supernatant at approximately 35-48 kD (Fig. 2d, e, f), whose intensity was substantially higher than any of the bands corresponding to CCA (A14). Since this band was reproducibly enriched in the extracellular supernatant, we hypothesized that the corresponding protein(s) might contain naturally efficient signal peptides that enable strong export capacity in *E. coli*. Therefore, this band was excised from the SDS-PAGE gel and subjected to LC–MS identification (Section 2.4). The identified proteins are listed in Supplementary Table S2. The higher the score, the more reliable the result. The N-terminal amino acid sequence of the top ten proteins from the identification results were examined for signal peptide possibility and cleavability using the Signal P server (version 6).

Importantly, the appearance of this prominent extracellular band was strongly dependent on the presence of an N-terminal signal peptide fusion. In the control strain expressing A14 without a signal peptide, this band was barely detectable in the culture supernatant. This signal peptide-dependent pattern suggests that the increased extracellular protein level and activity are mainly associated with improved translocation mediated by the engineered signal peptides, rather than being dominated by nonspecific release due to extensive cell lysis. Based on the MS results, six signal peptides were identified, including SP_*ompF*_, SP_*yncE*_, SP_*cysP*_, SP_*ompA*_, SP_*efeO*_ and SP_*potD*_, and were added to the N-terminus of A14, respectively (Fig. 2c). Despite a significant increase in the relative expression level of the enzyme (Fig. 2f), the addition of these signal peptides failed to confer a substantial improvement in secreted enzyme activity. The extracellular activity of all recombinant strains was only marginally enhanced, ranging from 1.04 to 1.07-fold that of the control (Fig. 2i).

Several signal peptides in Fig. 2 were found to favorably promote the secretion of the A14 protein. Among them, the signal peptide SP_AsPGA_ demonstrated superior performance by concurrently enhancing both the secretory efficiency and the functional activity of the enzyme. Consequently, SP_AsPGA_ was selected for subsequent investigations due to these dual advantages.

### 3.2 Site-directed mutagenesis of SP_AsPGA_ to enhance the secretion of protein A14

**Fig. 3:**
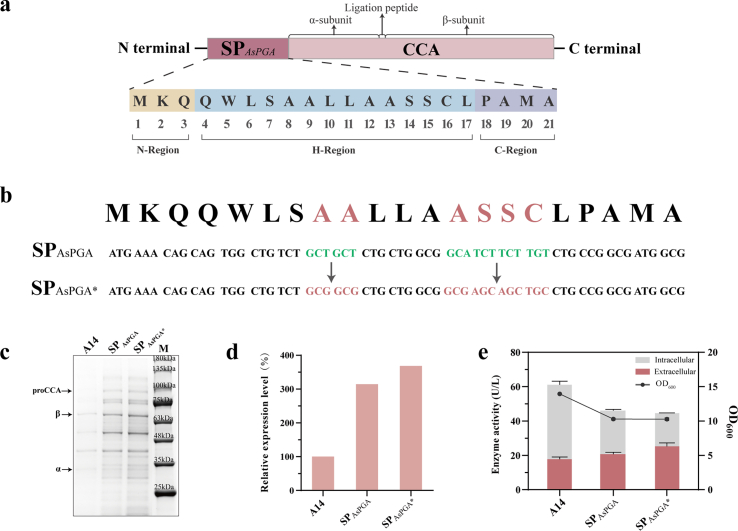
Systematic evaluation the mutants of SP_AsPGA_ by codon optimization. (a) The respective amino acid compositions of N-, H-, and C-regions of SP_AsPGA_. (b) The comparison of sequences before and after codon optimization of SP_AsPGA_. (c–e) The SDS-PAGE analysis of culture medium supernatant (c), relative extracellular expression levels (d), and intra- and extracellular enzyme activities (e) of strains expressing A14 fused to before and after codon optimization of SP_AsPGA_.

*In silico* analysis with SignalP predicted that SP_AsPGA_ is a 21-amino-acid peptide, with the respective amino acid compositions of its N-, H-, and C-regions detailed in Fig. 3a. To enhance its heterologous expression efficiency in *E. coli*, the native signal peptide SP_AsPGA_ was subjected to codon optimization. The optimization was mainly focused on eliminating codons that are rare in *E. coli* and replacing them with synonymous codons preferred by *E. coli*. In the native SP_AsPGA_ sequence derived from *Achromobacter* sp. CCM 4824, several codons with low usage frequency in *E. coli* were present, including codons encoding alanine and serine. Such rare codons may limit translation efficiency by imposing demand on low-abundance tRNAs, thereby causing transient ribosome pausing and reduced elongation efficiency. After optimization, these codons were systematically replaced by high-frequency synonymous codons (e.g., alanine codons were shifted toward *E. coli*-preferred GCG, and serine codons toward AGC) (Fig. 3b). The codon-optimized mutant SP_AsPGA∗_-A14 fusion exihibited 3.68- and 1.17-fold increase in relative expression level over A14 and SP_AsPGA_-A14 fusion, respectively (Fig. 3c and d), and also achieved 1.6- and 1.14-fold higher extracellular enzyme activity (Fig. 3e).

**Fig. 4:**
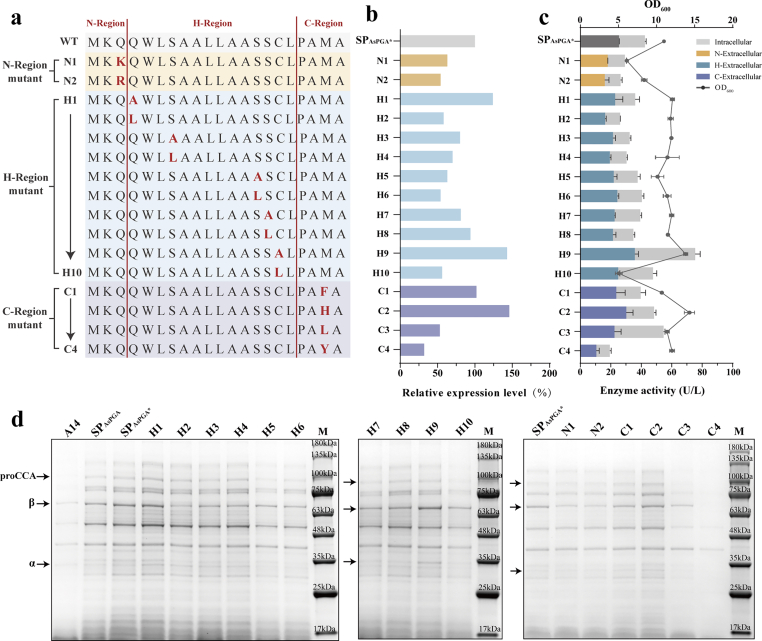
Systematic evaluation the mutants of SP_AsPGA_ by site-directed mutagenesis. (a) The amino acid sequences of the SP_AsPGA∗_ and its mutants. (b–d) The SDS-PAGE analysis of culture medium supernatant (d), relative extracellular expression levels (b), and intra- and extracellular enzyme activities (c) of strains expressing A14 fused to SP_AsPGA∗_ and its mutants.

The H-region (hydrophobic core) of a signal peptide is a critical determinant for protein secretion through the guarded gate—SecA/SecYEG. Its recognition by SecA depends on two key biophysical properties: hydrophobicity and helical propensity. Upon binding to the hydrophobic groove of SecA, the signal peptide undergoes a disorder-to-helix conformational transition [22]. This induced helical structure is essential for stimulating the ATPase activity of SecA, thereby initiating the translocation process [23]. Optimizing the hydrophobicity of the H-region of SP_AsPGA∗_ is expected to enhance the secretion efficiency of A14. We systematically substituted each of the five amino acids (Q4, S7, S14, S15, and C16) within the H-domain of SP_AsPGA∗_ with alanine (A) and leucine (L), respectively (Fig. 4a). The strain H9 contained mutant C16A demonstrated 6.26- and 1.43-fold increase in relative expression level over A14 and SP_AsPGA∗_-A14 fusion, respectively (Fig. 4b and d), and also exhibited 2- and 1.41-fold higher extracellular enzyme activity (Fig. 4c). The total enzyme activity of this mutant is 1.23- and 1.69-fold increase over A14 and SP_AsPGA∗_-A14 fusion, respectively (Fig. 4c).

A net charge of at least +1 in the N-region is a critical determinant for efficient recombinant protein export [10]. Furthermore, the optimal charge density for maximal secretion efficiency appears to be signal peptide-specific. We attempted to increase the secretion of A14 by substituting the resident amino acid at position 3 in the n-region with a basic residue—either lysine (K) or arginine (R) (Fig. 4a). The relative expression level of A14, as well as the extracellular enzyme activity and total enzyme activity, all decreased in the N1 and N2 mutant strains compared to SP_AsPGA∗_-A14 fusion (Fig. 4b and c). Moreover, the mutants showed a negative impact on growth (Fig. 4c). This suggests that increasing N-region positive charge is not universally beneficial and may depend on the specific signal peptide–passenger compatibility. Although positively charged residues in the N-region are generally considered beneficial for Sec-dependent targeting, the N1 and N2 variants (introducing an additional K or R) showed reduced expression and extracellular activity in our system. This result highlights the strong context dependence of signal peptide function. An increase in N-region positive charge may strengthen electrostatic interactions with the membrane, potentially altering insertion geometry or residence time at the membrane interface. In the specific N-terminal sequence context of CCA, such effects may reduce the formation of a productive SecA–preprotein complex or lead to transient stalling at the SecYEG translocon, ultimately decreasing translocation efficiency and soluble expression.

The efficiency of protein secretion is critically dependent on the precise cleavage of the signal peptide, an event mediated by signal peptidase. This enzyme recognizes and hydrolyzes the C-region. Due to its well-defined consensus pattern, the C-region constitutes a highly amenable and unambiguous target for rational optimization. Position 20 in C-region favoured large amino acids (i.e. phenylalanine, tyrosine, leucine and histidine) for efficient processing [10]. To enhance A14 secretion, we employed a rational design strategy by mutating the c-region at position 20 to canonical large residues: phenylalanine (F), tyrosine (Y), leucine (L), or histidine (H) (Fig. 4a). The C2 strain exihibited 5.37- and 1.46-fold increase in relative expression level over A14 and SP_AsPGA∗_-A14 fusion, respectively (Fig. 4b), and also achieved 2.36- and 1.18-fold higher extracellular enzyme activity (Fig. 4c). However, the total enzyme activity of C2 was lower than that of A14, being only 0.79-fold that of A14, while it was 1.08-fold higher than that of SP_AsPGA∗_-A14. Despite the C3 mutant exhibiting the highest total enzyme activity among the four mutants—1.22-fold greater than the SP_AsPGA∗_-A14 fusion strain—its total activity was still 10% lower than that of the original A14 strain (Fig. 4c). Furthermore, both the extracellular enzyme activity and the relative expression level of the C3 mutant were reduced compared to A14 (Fig. 4b and c).

The H9 signal peptide mutant exhibited the most favorable phenotype among all the signal peptide mutants. It significantly increased the extracellular expression and activity of A14. In addition to these secretion-related improvements, the H9 strain also showed superior cell growth and higher total enzymatic activity compared to other mutants.

To evaluate whether synonymous and/or amino-acid substitutions in the signal peptide region could potentially affect translation efficiency through altered mRNA folding, the mRNA secondary structures corresponding to the signal peptide coding sequences were predicted using RNAfold (ViennaRNA package) (Fig. S9). The predicted minimum free energy (MFE) of the native SP_AsPGA_ was −51.10 kcal/mol, whereas the optimized SP_AsPGA∗_ and the variant H9 (C16A) exhibited more negative MFEs of −53.60 and −56.00 kcal/mol, respectively. These results indicate that the introduced sequence modifications increased the thermodynamic stability of local mRNA secondary structures around the N-terminal signal peptide region.

Notably, changes in mRNA folding near the translation initiation region and early coding sequence have been reported to modulate ribosome accessibility and translation initiation/early elongation kinetics, thereby influencing protein expression outcomes. In this context, the observed decrease in MFE suggests that the engineered variants may alter the local unfolding barrier experienced by the ribosome during early translation, which could contribute to the differences in expression and secretion efficiency.

### 3.3 C16A Stabilizes Signal Peptide Conformation to Promote Protein Translocation via SecA

**Fig. 5:**
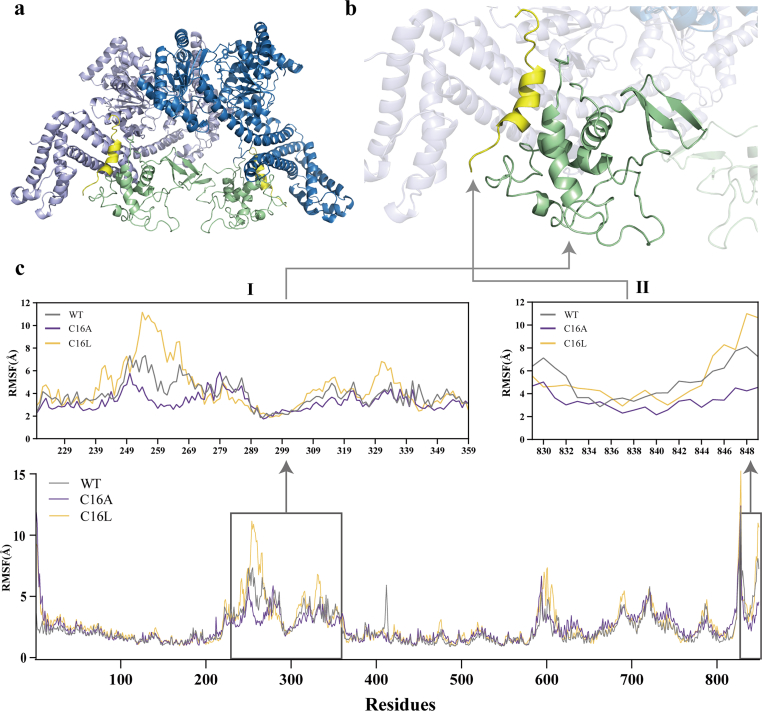
Dynamic analysis of signal peptide-SecA complex. (a) 3D structure of the signal peptide-SecA complex. (b) Signal peptide (yellow) and its regulatory binding region (green) within signal peptide-SecA complex. (c) Conformational fluctuation alterations in key functional regions of the signal peptide-SecA complex induced by C16A/C16L mutations (WT: grey; C16A: purple; C16L: yellow).

To further elucidate the impact of C16A and C16L mutations on the secretion level of A14, we employed molecular dynamics simulations to compare the conformational changes in both mutants and the wild-type. The binding of signal peptides to SecA is shown in Fig. 5a and b. Root mean square fluctuation (RMSF) analysis revealed that the C16A mutation in H9 reduced the overall structural fluctuation of the fusion of signal peptide and A14 (Fig. 5c). Specifically, the fluctuations in Region I (residues 220–359, Fig. 5c, shown in purple), which is in the PBD domain [24], could regulate signal peptide binding, and in the signal peptide itself (residues 829–849, Fig. 5c, shown in purple) were significantly diminished, enabling a more stable binding conformation throughout the dynamics process. In contrast, the C16L mutation in H10 increased fluctuations in the same regions, resulting in markedly greater flexibility and a relatively unstable conformation (Fig. 5a, shown in yellow). The binding energy calculations indicated that the binding energies of H9 and H10 model were nearly identical (−38.8 kcal·mol^−1^ and -38.6 kcal·mol^−1^, respectively) and both were superior to that of the wild-type (−33.6 kcal·mol^−1^). This suggests that enhanced hydrophobic interactions may facilitate the binding between the signal peptide and SecA. In summary, the C16A mutation accelerates upstream targeting and delivery by reducing conformational fluctuations and stabilizing the structural domain, whereas the increased flexibility induced by C16L may delay the delivery process. These findings further demonstrate that the stable conformation formed between the signal peptide and the SecA system is critical for the efficient translocation of the target protein.

## 4 Conclusion

The CCA mutant A14 derived from *Bosea* sp. OK403, which does not contain signal peptide, is an intracellular enzyme that demonstrates significant potential for hydrolyzing a broad spectrum of β-lactam antibiotics [7]. To enhance the heterologous production of A14 in *E. coli*, a systematic engineering strategy was employed, focusing on the critical role of the signal peptide. First, we adopted a multi-strategy approach for signal peptide selection. Three distinct classes of signal peptides were tested, including: (i) well-characterized, commonly used signal peptides; (ii) native signal peptides derived from penicillin G acylases (PGAs) of various bacterial origins; and (iii) novel signal peptides identified through mass spectrometry-based proteomic screening. The initial screening efforts identified the native signal peptide SP_AsPGA_ as a superior candidate for directing A14 through the Sec-dependent translocation pathway.

Driven by this hypothesis, we turned to rational design of SP_AsPGA_. By performing site-directed mutagenesis across its N-, H-, and C-regions, we aimed to optimize its structure and function. Among the engineered variants, the H9 strain, carrying the C16A mutation, achieved a 6.26-fold increase in relative expression compared to the native A14 and a 1.43-fold increase over the initial SP_AsPGA∗_-A14 fusion. More critically, this elevated expression directly translated to superior functional output, as evidenced by a 2.0-fold and 1.41-fold increase in extracellular enzyme activity relative to the same controls, respectively. Furthermore, the total enzyme activity of the H9 strain was elevated by 1.23-fold and 1.69-fold over the A14 and SP_AsPGA∗_-A14 benchmarks. The H9 mutant emerged as the most promising, demonstrating that a tailored signal peptide sequence can simultaneously enhance extracellular expression, enzymatic activity, and host cell fitness. This multifaceted improvement indicates that an optimized signal peptide not only facilitates more efficient translocation but also alleviates the cellular burden associated with heterologous protein production.

To elucidate the mechanistic basis for these observations, molecular dynamics simulations were conducted on key mutants. The analysis revealed a structure-function relationship wherein superior performance, as seen in the C16A variant, correlates with reduced conformational fluctuations in critical regions, thereby stabilizing the signal peptide's interaction with SecA. In contrast, the C16L mutation, which increased flexibility, was predicted to delay delivery. These computational insights confirm that the stability of the signal peptide-SecA complex is a critical determinant for efficient protein translocation.

In conclusion, our findings demonstrate that the functional secretion of A14 in *E. coli* can be significantly enhanced through signal peptide engineering. The success of the H9 mutant exemplifies the efficacy of combining signal peptide mining and screening with rational mutagenesis. Furthermore, we establish that the stabilization of the signal peptide's conformation, particularly its binding interface with SecA, is a key mechanism underpinning enhanced translocation efficiency. This study provides a comprehensive strategy for optimizing heterologous protein secretion, highlighting the signal peptide not merely as a passive leader, but as a central and tunable component in the recombinant expression.

## CRediT authorship contribution statement

**Xinyi Ren:** Writing – review & editing, Writing – original draft, Visualization, Validation, Methodology, Investigation, Formal analysis, Data curation. **Zhiying Yang:** Writing – review & editing, Validation, Methodology, Data curation. **Siyu Chen:** Writing – review & editing, Validation, Methodology, Data curation. **Chunxiang Pu:** Writing – review & editing, Validation, Software, Methodology. **Huiying Wang:** Validation, Methodology, Investigation. **Jinlong Li:** Visualization, Validation, Software. **Qichen Cao:** Methodology, Data curation. **Xuyang Huang:** Visualization, Validation. **Gang Fu:** Methodology, Investigation. **Bo Yuan:** Supervision, Resources, Project administration. **Jianmei Luo:** Writing – review & editing, Validation, Project administration, Funding acquisition. **Zhoutong Sun:** Supervision, Resources, Project administration, Funding acquisition. **Huina Dong:** Writing – review & editing, Validation, Resources, Project administration, Funding acquisition. **Dawei Zhang:** Writing – review & editing, Project administration, Funding acquisition.

## Declaration of competing interest

The authors declare that they have no known competing financial interests or personal relationships that could have appeared to influence the work reported in this paper.
