# Supplementary material for: Systematic mining and engineering of signal peptides to achieve efficient secretion of cephalosporin C acylase in Escherichia coli
Source: Synth Syst Biotechnol. 2026 Mar 24;13:508–16. doi: 10.1016/j.synbio.2026.03.006 (PMC13049609; doi:10.1016/j.synbio.2026.03.006)
Supplement: Multimedia component 2 [file mmc2.doc]

**Systematic Mining and Engineering of Signal Peptides to Achieve Efficient Secretion of Cephalosporin C Acylase in *Escherichia coli***

Xinyi Ren^1^, ^2, †^, Zhiying Yang^1^, ^2, †^, Siyu Chen^1^, ^2, †^, Chunxiang Pu^2, †^, Huiying Wang^2^, Jinlong Li^2^, Qichen Cao^1^, ^2, 3^, Xuyang Huang^2^, Gang Fu^1, 2, 3^, Bo Yuan^1, 2, 3, 4^, Jianmei Luo^1, *^, Zhoutong Sun^1, 2, 3, 4, *^, Huina Dong^1^, ^2, 3, *^, and Dawei Zhang^1, 2, 3, 4, *^

^1^ Tianjin University of Science and Technology, Tianjin, China

^2^ Tianjin Institute of Industrial Biotechnology, Chinese Academy of Sciences, Tianjin, China

^3^ University of Chinese Academy of Sciences, Beijing, China

^4^ State Key Laboratory of Engineering Biology for Low-Carbon Manufacturing, Tianjin Institute of Industrial Biotechnology, Chinese Academy of Sciences, Tianjin, China

^†^These authors equally contributed to this work.

*Correspondence: luojianmei@tust.edu.cn, [sunzht@tib.cas.cn,](mailto:luojianmei@tust.edu.cn,) dong_hn@tib.cas.cn, [zhang_dw@tib.cas.cn](mailto:zhang_dw@tib.cas.cn,).

Table S1. Plasmids Used in This Study

| Plasmids | Genotype or relevant characteristics | Sources |
| --- | --- | --- |
| pET28a-A14 | CA from *Bosea sp*. OK403 with double mutations L159S/A419V |  |
| pET28a-SP*_pelB_*-A14 | pET28a-A14 derivative, SP*_pelB_* | This work |
| pET28a-SP*_lamB_*-A14 | pET28a-A14 derivative, SP*_lamB_* | This work |
| pET28a-SP*_phoA_*-A14 | pET28a-A14 derivative, SP*_phoA_* | This work |
| pET28a-SP*_malE_*-A14 | pET28a-A14 derivative, SP*_malE_* | This work |
| pET28a-SP*_AsPGA_*-A14 | pET28a-A14 derivative, SP*_AsPGA_* | This work |
| pET28a-SP*_AxPGA_*-A14 | pET28a-A14 derivative, SP*_AxPGA_* | This work |
| pET28a-SP*_AfPGA_*-A14 | pET28a-A14 derivative, SP*_AfPGA_* | This work |
| pET28a-SP*_EcPGA_*-A14 | pET28a-A14 derivative, SP*_EcPGA_* | This work |
| pET28a-SP*_ompF_*-A14 | pET28a-A14 derivative, SP*_ompF_* | This work |
| pET28a-SP*_yncE_*-A14 | pET28a-A14 derivative, SP*_yncE_* | This work |
| pET28a-SP*_cysP_*-A14 | pET28a-A14 derivative, SP*_cysP_* | This work |
| pET28a-SP*_ompA_*-A14 | pET28a-A14 derivative, SP*_ompA_* | This work |
| pET28a-SP*_efeO_*-A14 | pET28a-A14 derivative, SP*_efeO_* | This work |
| pET28a-SP*_potD_*-A14 | pET28a-A14 derivative, SP*_potD_* | This work |
| pET28a-SP*_AsPGA_**-A14 | pET28a-SP*_AsPGA_*-A14 derivative, SP*_AsPGA_** | This work |
| pET28a-H1-A14 | pET28a-SP*_AsPGA_**-A14 derivative, SP*_AsPGA_** with mutation Q4A | This work |
| pET28a-H2-A14 | pET28a-SP*_AsPGA_**-A14 derivative, SP*_AsPGA_** with mutation Q4L | This work |
| pET28a-H3-A14 | pET28a-SP*_AsPGA_**-A14 derivative, SP*_AsPGA_** with mutation S7A | This work |
| pET28a-H4-A14 | pET28a-SP*_AsPGA_**-A14 derivative, SP*_AsPGA_** with mutation S7L | This work |
| pET28a-H5-A14 | pET28a-SP*_AsPGA_**-A14 derivative, SP*_AsPGA_** with mutation S14A | This work |
| pET28a-H6-A14 | pET28a-SP*_AsPGA_**-A14 derivative, SP*_AsPGA_** with mutation S14L | This work |
| pET28a-H7-A14 | pET28a-SP*_AsPGA_**-A14 derivative, SP*_AsPGA_** with mutation S15A | This work |
| pET28a-H8-A14 | pET28a-SP*_AsPGA_**-A14 derivative, SP*_AsPGA_** with mutation S15L | This work |
| pET28a-H9-A14 | pET28a-SP*_AsPGA_**-A14 derivative, SP*_AsPGA_** with mutation C16A | This work |
| pET28a-H10-A14 | pET28a-SP*_AsPGA_**-A14 derivative, SP*_AsPGA_** with mutation C16L | This work |
| pET28a-N1-A14 | pET28a-SP*_AsPGA_**-A14 derivative, SP*_AsPGA_** with mutation Q3K | This work |
| pET28a-N2-A14 | pET28a-SP*_AsPGA_**-A14 derivative, SP*_AsPGA_** with mutation Q3R | This work |
| pET28a-C1-A14 | pET28a-SP*_AsPGA_**-A14 derivative, SP*_AsPGA_** with mutation M20F | This work |
| pET28a-C2-A14 | pET28a-SP*_AsPGA_**-A14 derivative, SP*_AsPGA_** with mutation M20H | This work |
| pET28a-C3-A14 | pET28a-SP*_AsPGA_**-A14 derivative, SP*_AsPGA_** with mutation M20L | This work |
| pET28a-C4-A14 | pET28a-SP*_AsPGA_**-A14 derivative, SP*_AsPGA_** with mutation M20Y | This work |

Table S2. Strains Used in This Study

| Strains | Genotype or relevant characteristics | Sources |
| --- | --- | --- |
| *E. coli* DH5α | Host for construction of plasmids | Invitrogen |
| *E. coli* BL21(DE3) | Host for expression of enzymes | Invitrogen |
| A14 | Initial strain containing pET28a-A14 plasmid |  |
| A14-pelB | Initial strain containing pET28a-SP*_pelB_*-A14 plasmid | This work |
| A14-malE | Initial strain containing pET28a-SP*_malE_*-A14 plasmid | This work |
| A14-lamB | Initial strain containing pET28a-SP*_lamB_*-A14 plasmid | This work |
| A14-phoA | Initial strain containing pET28a-SP*_phoA_*-A14 plasmid | This work |
| AsPGA | Initial strain containing pET28a-SP*_AsPGA_*-A14 plasmid | This work |
| AxPGA | Initial strain containing pET28a-SP*_AxPGA_*-A14 plasmid | This work |
| AfPGA | Initial strain containing pET28a-SP*_AfPGA_*-A14 plasmid | This work |
| EcPGA | Initial strain containing pET28a-SP*_EcPGA_*-A14 plasmid | This work |
| SP1 | Initial strain containing pET28a-SP*_ompF_*-A14 plasmid | This work |
| SP2 | Initial strain containing pET28a-SP*_yncE_*-A14 plasmid | This work |
| SP3 | Initial strain containing pET28a-SP*_cysP_*-A14 plasmid | This work |
| SP4 | Initial strain containing pET28a-SP*_ompA_*-A14 plasmid | This work |
| SP5 | Initial strain containing pET28a-SP*_efeO_*-A14 plasmid | This work |
| SP6 | Initial strain containing pET28a-SP*_potD_*-A14 plasmid | This work |
| AsPGA* | Initial strain containing pET28a-SP*_AsPGA*_*-A14 plasmid | This work |
| H1 | Initial strain containing pET28a-H1-A14 plasmid | This work |
| H2 | Initial strain containing pET28a-H2-A14 plasmid | This work |
| H3 | Initial strain containing pET28a-H3-A14 plasmid | This work |
| H4 | Initial strain containing pET28a-H4-A14 plasmid | This work |
| H5 | Initial strain containing pET28a-H5-A14 plasmid | This work |
| H6 | Initial strain containing pET28a-H6-A14 plasmid | This work |
| H7 | Initial strain containing pET28a-H7-A14 plasmid | This work |
| H8 | Initial strain containing pET28a-H8-A14 plasmid | This work |
| H9 | Initial strain containing pET28a-H9-A14 plasmid | This work |
| H10 | Initial strain containing pET28a-H10-A14 plasmid | This work |
| N1 | Initial strain containing pET28a-N1-A14 plasmid | This work |
| N2 | Initial strain containing pET28a-N2-A14 plasmid | This work |
| C1 | Initial strain containing pET28a-C1-A14 plasmid | This work |
| C2 | Initial strain containing pET28a-C2-A14 plasmid | This work |
| C3 | Initial strain containing pET28a-C3-A14 plasmid | This work |
| C4 | Initial strain containing pET28a-C4-A14 plasmid | This work |

Table S3. Primers Used in This Study

| **Name** | | **Primers** | **Notes** |
| --- | --- | --- | --- |
| AsPGA-F | GCTCTGCTGGCGGCATCTTCTTGTCTGCCGGCGATGGCGACCACCGATGCAGA | | Construction of signal peptides mined from PGA plasmids |
| AsPGA-R | GAAGATGCCGCCAGCAGAGCAGCAGACAGCCACTGCTGTTTCATggatccgcgaccca | |
| AxPGA-F | GCCATCCTGGCGGCCTGTGCGGCCGTGGGCGCTCATGCCCAGGCCACCACCGATGCAGATC | |
| AxPGA-R | GCCGCACAGGCCGCCAGGATGGCGGCCGACAACCATTGCTGCTTCATggatccgcgacccatttgct | |
| AfPGA-F | CTTGTGGCCGCTGGTTTGATCTTGGGTTGGGCGGGGGCACCGACCCACGCGACCACCGATGCAGA | |
| AfPGA-R | GATCAAACCAGCGGCCACAAGCCCGGTACGAACAAGCCCTTTCTGCATggatccgcgacccatttgct | |
| EcPGA-F | gaactgtgttactgcttccctgatgtattattggagcttacctgcactggctACCACCGATGCAGA | |
| EcPGA-R | cagggaagcagtaacacagttcacgatcatacgatttctatttttcatggatccgcgaccca | |
| ompF(SP1)-F | ggcagtgatcgtccctgctctgttagtagcaggtactgcaaacgctACCACCGATGCAGATCGTGATG | | Construction of identification and screening of signal peptides of proteins via mass spectrometry plasmids |
| ompF(SP1)-R | taacagagcagggacgatcactgccagaatattgcgcttcatcatggatccgcgacccatttgctgtcca | |
| yncE(SP2)-F | tgcgtggttcattactgttaggttcattgcttgttgcttcatcattcagtacgcaggccACCACCGATGCAGATCGTGATG | |
| yncE(SP2)-R | cctaacagtaatgaaccacgcaggcgcgatgaaaacagatgacgtaaatgcatggatccgcgacccatttgctgtcca | |
| cysP(SP3)-F | ctcactcgcgctggtcgcttctctgctgctggcgggccatgtacaggcaACCACCGATGCAGATCGTGATG | |
| cysP(SP3)-R | agcgaccagcgcgagtgagttctttttcagtaagttaacggccatggatccgcgacccatttgctgtcca | |
| ompA(SP4)-F | gcgattgcagtggcactggctggtttcgctaccgtagcgcaggccACCACCGATGCAGATCGTGATG | |
| ompA(SP4)-R | aaaccagccagtgccactgcaatcgcgatagctgtctttttcatggatccgcgacccatttgctgtcc | |
| efeO(SP5)-F | gcagttgagcgtggctgcgctgttttcttctgcttttatggctaacgccACCACCGATGCAGATCGTGATG | |
| efeO(SP5)-R | agcgcagccacgctcaactgcaatgcgttacggcggaagttaatggtcatggatccgcgacccatttgctgtcca | |
| potD(SP6)-F | cctgctcgcggcgggtgctctggcactgggcatgagcgccgctcacgccACCACCGATGCAGATCGTGATG | |
| potD(SP6)-R | ccagagcacccgccgcgagcaggtggcgtgaccattttttcatggatccgcgacccatttgctgtcca | |
| pelB-F | CGACCGCTGCTGCTGGTCTGCTGCTCCTCGCTGCCCAGCCGGCGATGGCCATGACCACCGATGCAGATCGTGATGC | | Construction of commonly used signal peptides plasmids |
| pelB-R | GCAGCAGACCAGCAGCAGCGGTCGGCAGCAGGTATTTCATggatccgcgacccatttgctgtcc | |
| phoA-F | cactggcactcttaccgttactgtttacccctgtgacaaaagccATGACCACCGATGCAGATCGTGATG | |
| phoA-R | acagtaacggtaagagtgccagtgcaatagtgctttgtttcatggatccgcgacccatttgctgtcc | |
| malE-F | ctcgcattatccgcattaacgacgatgatgttttccgcctcggctATGACCACCGATGCAGATCGTGATG | |
| malE-R | atcgtcgttaatgcggataatgcgaggatgcgtgcacctgtttttattttcatggatccgcgacccatttgctgtcc | |
| lamB-F | cttcctctggcggttgccgtcgcagcgggcgtaatgtctgctcaggcaATGACCACCGATGCAGATCGTGATG | |
| lamB-R | cgctgcgacggcaaccgccagaggaagtttgcgcagagtaatcatcatggatccgcgacccatttgctgtcca | |
| AsPGA*-F | CTGCGGCGCTGCTGGCGGCGAGCAGCTGCCTGCCGGCGATGGCGACCACCGATGCAGATCGTGA | | Construction of SP_AsPGA_ optimization plasmids |
| AsPGA*-R | TCGCCGCCAGCAGCGCCGCAGACAGCCACTGCTGTTTCATggatccgcgacccatttgctgtc | |
| Q4A-F | atccATGAAACAGGCGTGGCTGTCTGCGGCGCTGCTG | |
| Q4A-R | GCCGCAGACAGCCACGCCTGTTTCATggatccgcgacc | |
| Q4L-F | atccATGAAACAGCTGTGGCTGTCTGCGGCGCTGCTG | |
| Q4L-R | GCCGCAGACAGCCACAGCTGTTTCATggatccgcgacc | |
| S7A-F | CAGTGGCTGGCGGCGGCGCTGCTGGCGGCATC | |
| S7A-R | CAGCAGCGCCGCCGCCAGCCACTGCTGTTTCATggatc | |
| S7L-F | CAGTGGCTGCTGGCGGCGCTGCTGGCGGCATC | |
| S7L-R | CAGCAGCGCCGCCAGCAGCCACTGCTGTTTCATggatc | |
| S14A-F | TCTGCTGGCGGCGGCGAGCTGCCTGCCGGCGATGGC | |
| S14A-R | CAGGCAGCTCGCCGCCGCCAGCAGCGCCGCAG | |
| S14L-F | TCTGCTGGCGGCGCTGAGCTGCCTGCCGGCGATGGC | |
| S14L-R | CAGGCAGCTCAGCGCCGCCAGCAGCGCCGCAG | |
| S15A-F | GGCGGCGAGCGCGTGCCTGCCGGCGATGGCGACC | |
| S15A-R | CAGGCACGCGCTCGCCGCCAGCAGCGCCGCAG | |
| S15L-F | GGCGGCGAGCCTGTGCCTGCCGGCGATGGCGACC | |
| S15L-R | CAGGCACAGGCTCGCCGCCAGCAGCGCCGCAG | |
| C16A-F | GCGGCGAGCAGCGCGCTGCCGGCGATGGCGACCACC | |
| C16A-R | CATCGCCAGCGCGCTGCTCGCCGCCAGCAGCGCCGCAG | |
| C16L-F | GCGGCGAGCAGCCTGCTGCCGGCGATGGCGACCACC | |
| C16L-R | CATCGCCAGCAGGCTGCTCGCCGCCAGCAGCGCCGCAG | |
| Q3K-F | cgcggatccATGAAAAAACAGTGGCTGTCTGCGGCG | |
| Q3K-R | CAGACAGCCACTGTTTTTTCATggatccgcgacccatt | |
| Q3R-F | cgcggatccATGAAACGCCAGTGGCTGTCTGCGGCG | |
| Q3R-R | CAGACAGCCACTGGCGTTTCATggatccgcgacccatt | |
| M20F-F | CTGCCTGCCGGCGTTTGCGACCACCGATGCAGATCG | |
| M20F-R | GCATCGGTGGTCGCAAACGCCGGCAGGCAGCTGCTC | |
| M20H-F | CTGCCTGCCGGCGCATGCGACCACCGATGCAGATCG | |
| M20H-R | GCATCGGTGGTCGCATGCGCCGGCAGGCAGCTGCTC | |
| M20L-F | CTGCCTGCCGGCGCTGGCGACCACCGATGCAGATCG | |
| M20L-R | GCATCGGTGGTCGCCAGCGCCGGCAGGCAGCTGCTC | |
| M20Y-F | CTGCCTGCCGGCGTATGCGACCACCGATGCAGATCG | |
| M20Y-R | GCATCGGTGGTCGCATACGCCGGCAGGCAGCTGCTC | |


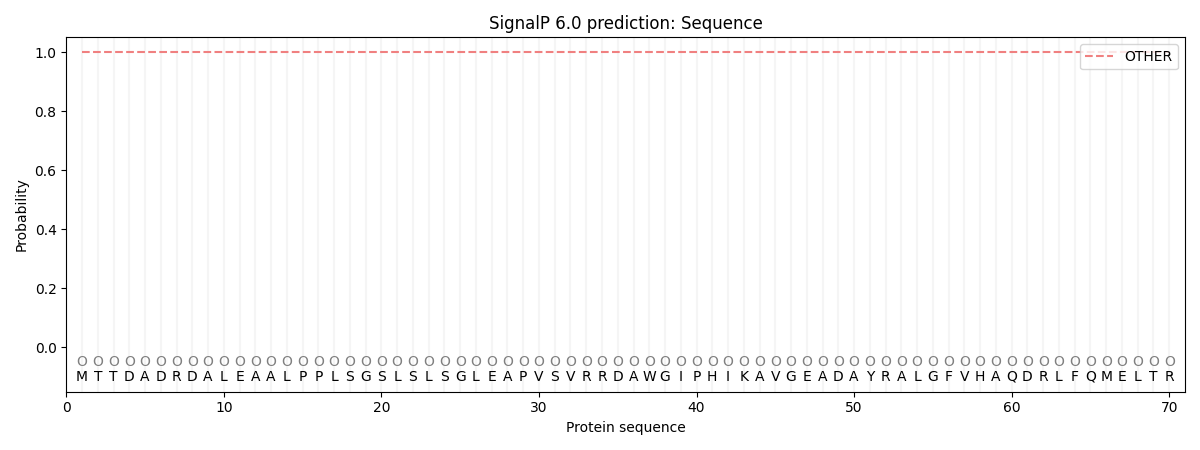


Fig. S1 Signal peptide prediction results of A14 sequence.


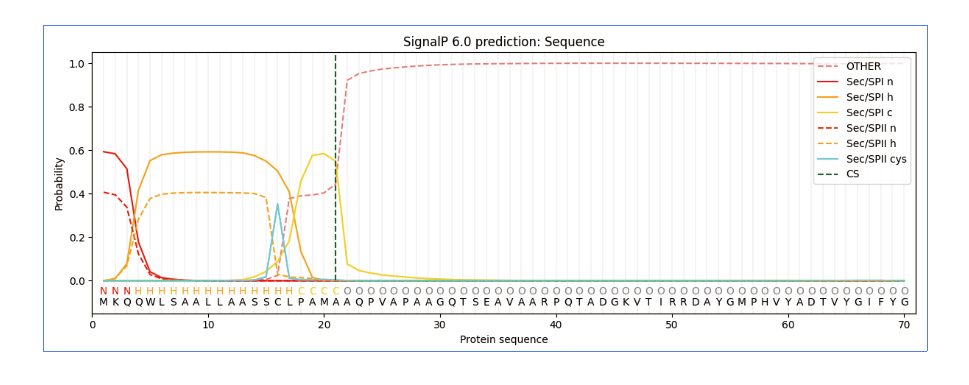


Fig. S2 Signal peptide prediction results of PGA sequence.


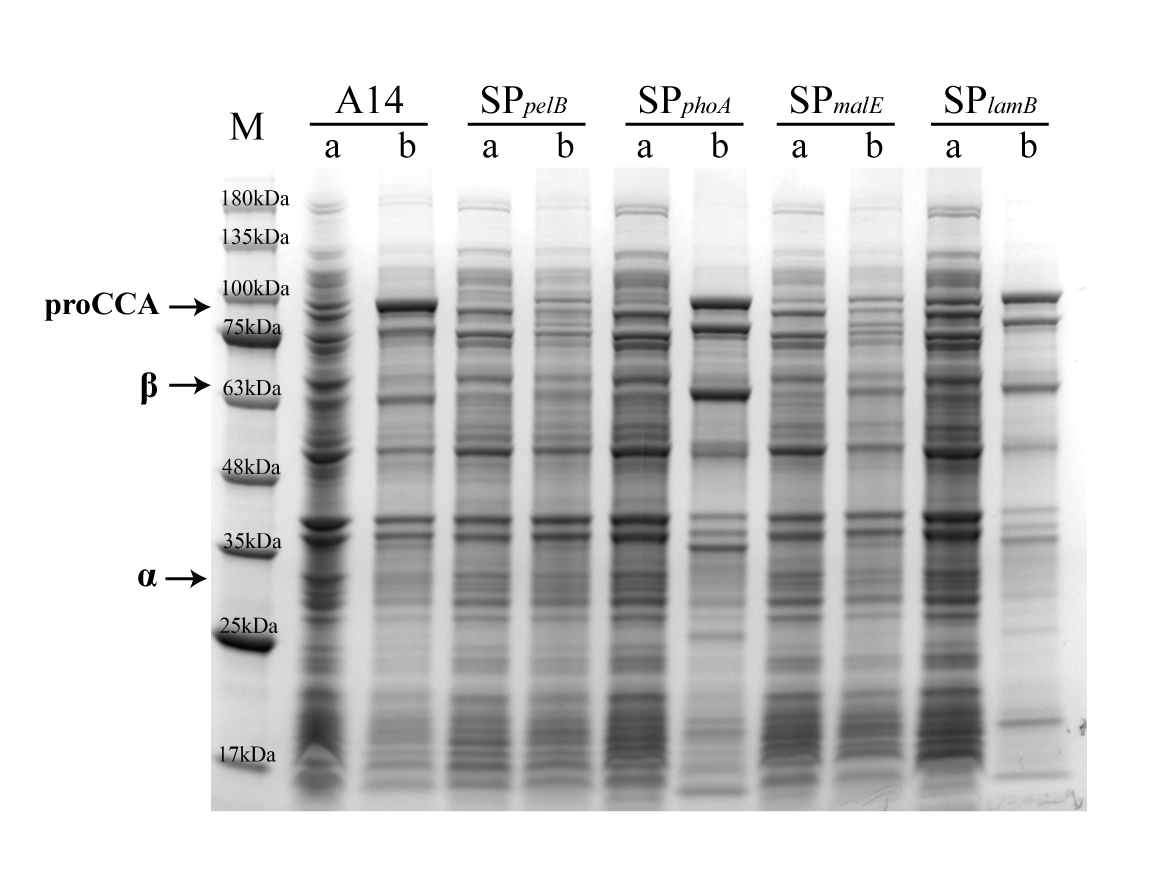


Fig. S3 Expression of the CCA enzyme with commonly used *E. coli* signal peptides in the soluble and insoluble fractions. M, protein marker; “a” indicated the soluble fractions; “b” indicated the insoluble fractions;


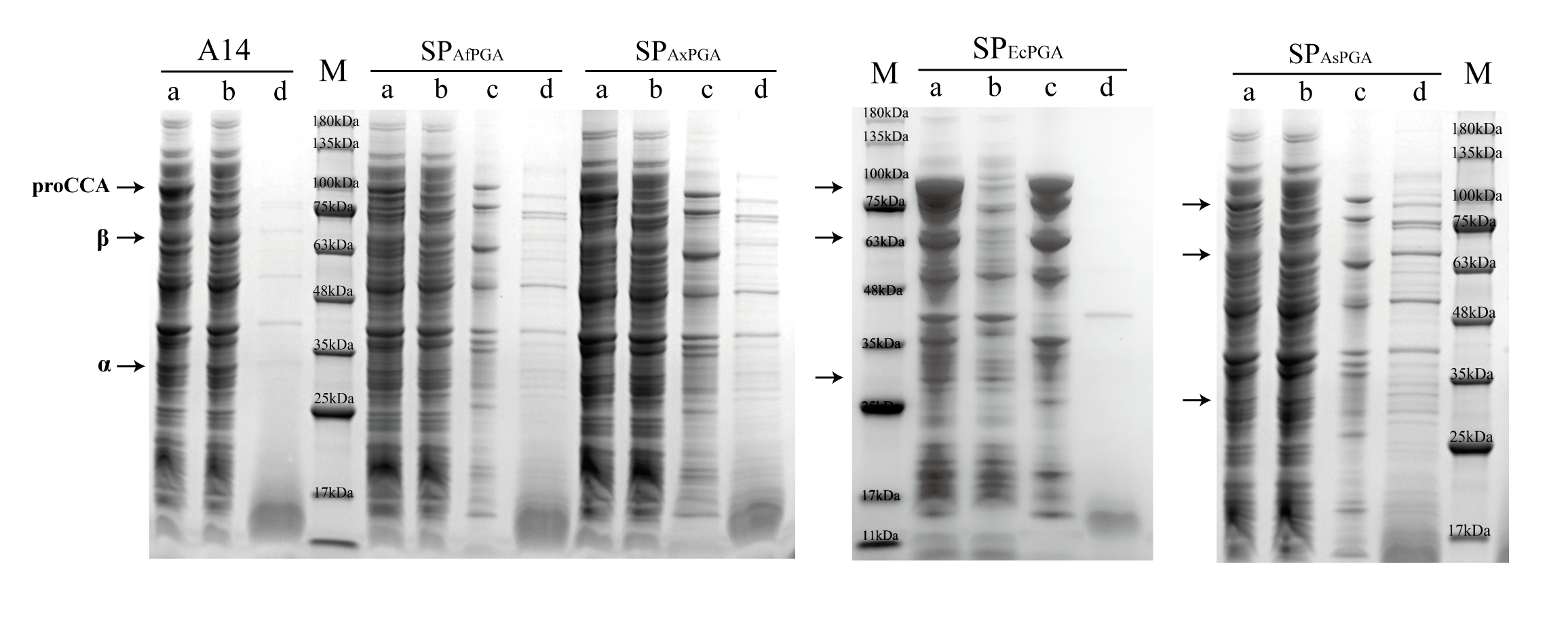


Fig. S4 Expression of the CCA enzyme with native signal peptides from penicillin G acylase. M, protein marker; “a” indicated the total cellular proteins; “b” indicated the soluble fractions; “c” indicated the insoluble fractions; “d” indicated the protein components in the culture supernatant.


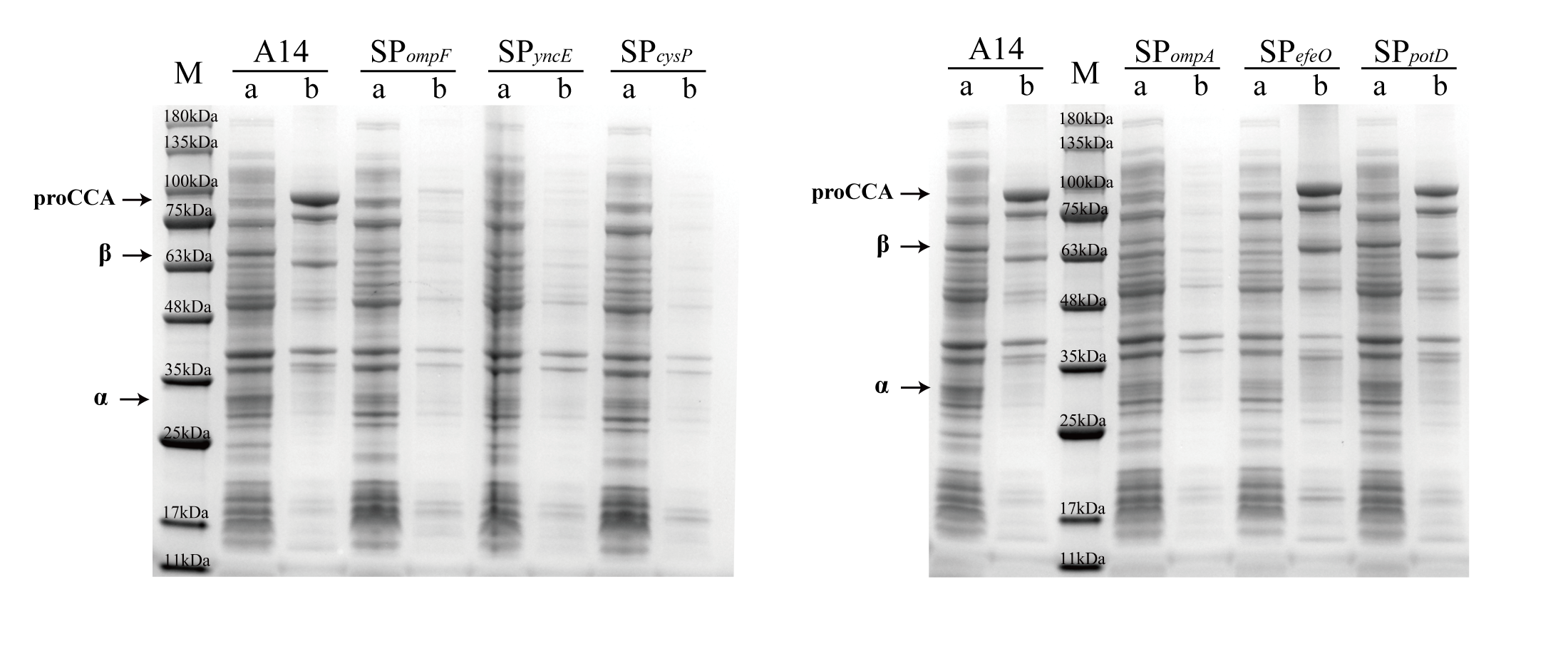


Fig. S5. Expression of the CCA enzyme with signal peptides screened by proteomic identification. M, protein marker; “a” indicated the soluble fractions; “b” indicated the insoluble fractions;


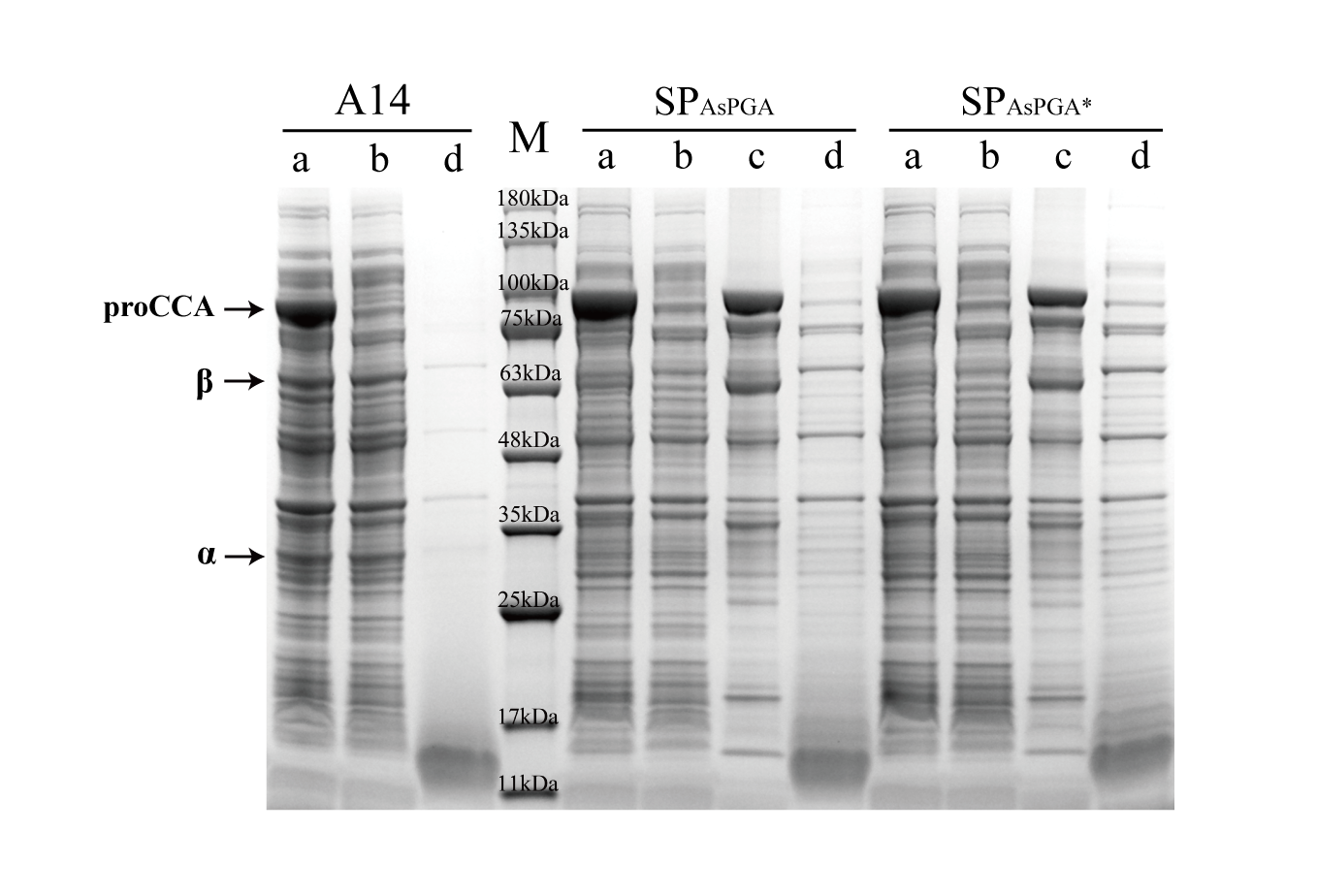


Fig. S6. Expression of the CCA enzyme after codon optimization of SP_AsPGA_. M, protein marker; “a” indicated the total cellular proteins; “b” indicated the soluble fractions; “c” indicated the insoluble fractions; “d” indicated the protein components in the culture supernatant.


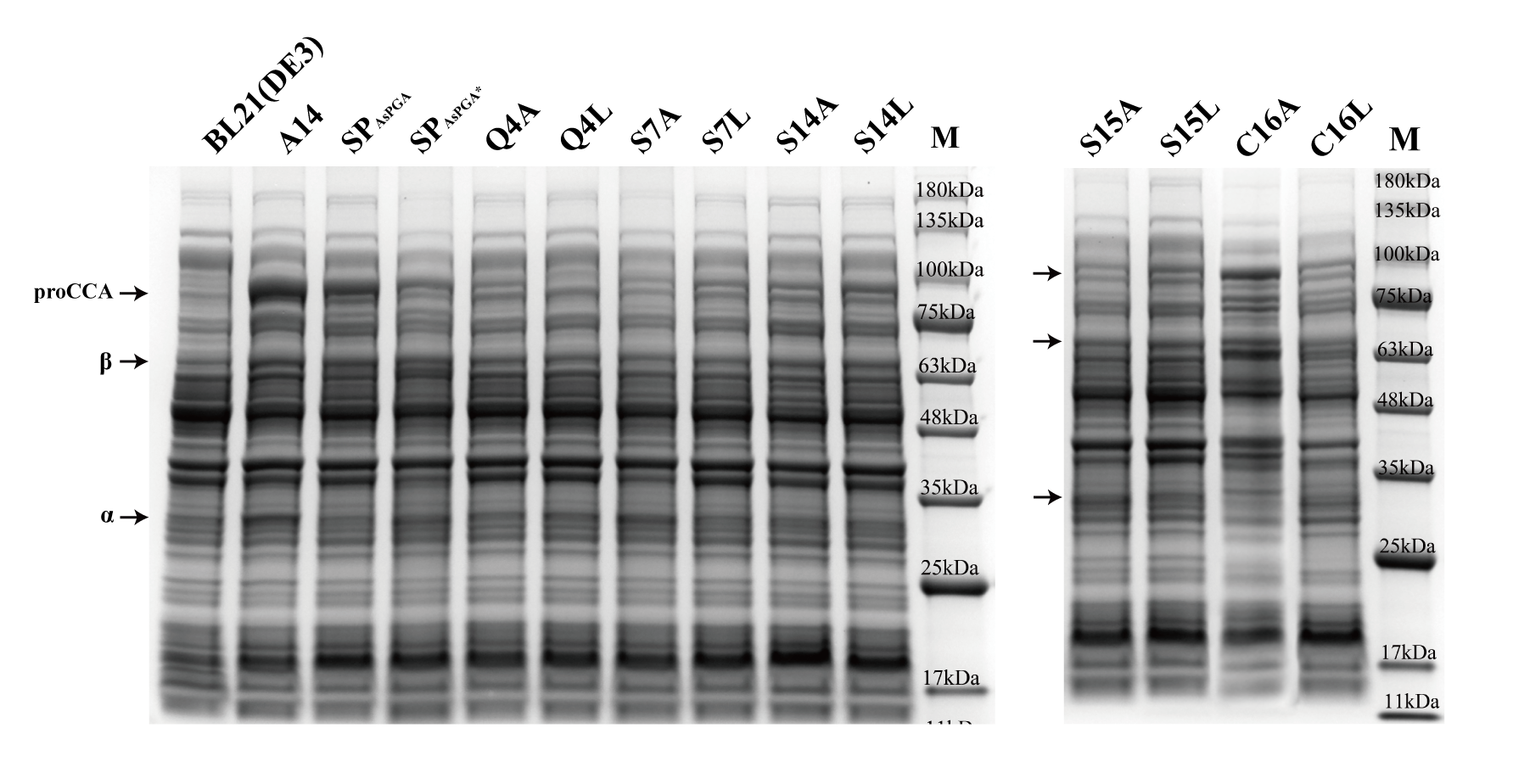


Fig. S7. Total protein expression of the CCA enzyme after mutations in the H-region of SP_AsPGA*_.


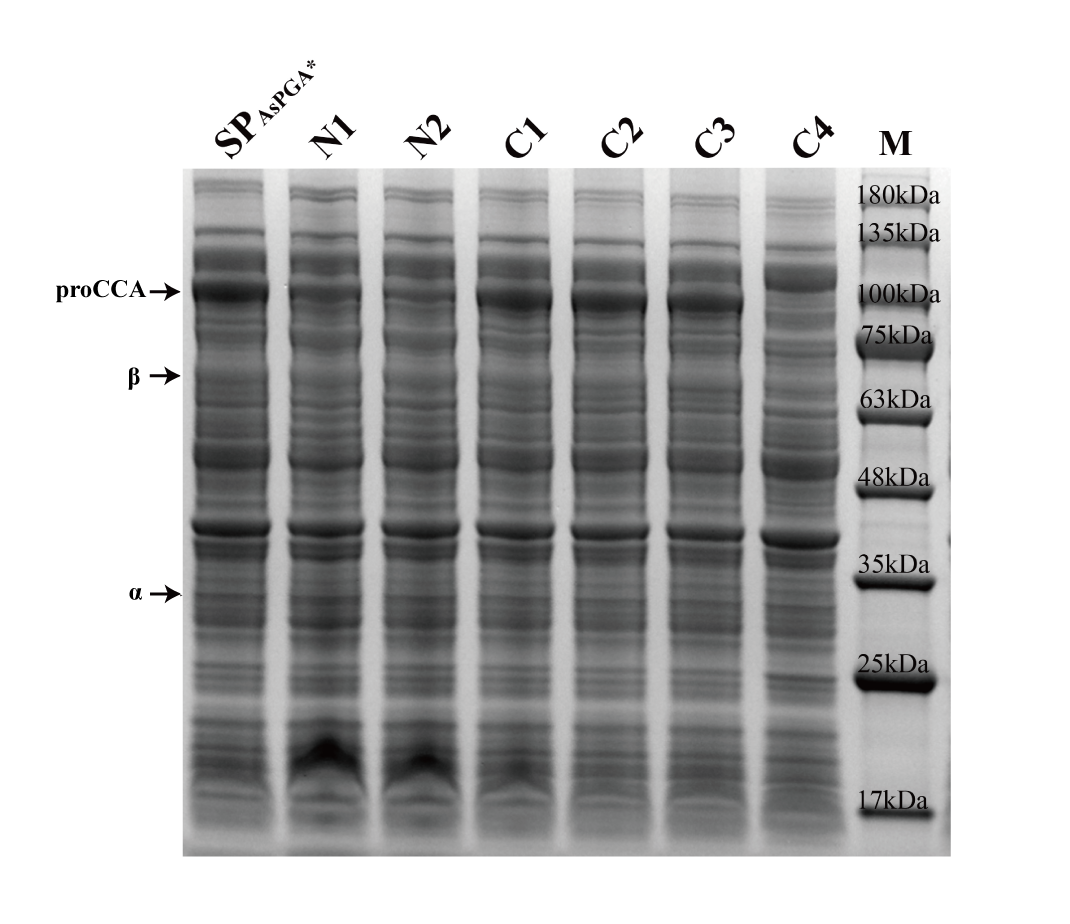


Fig. S8 Total protein expression of the CCA enzyme after mutations in the N- and C- region of SP_AsPGA*_.
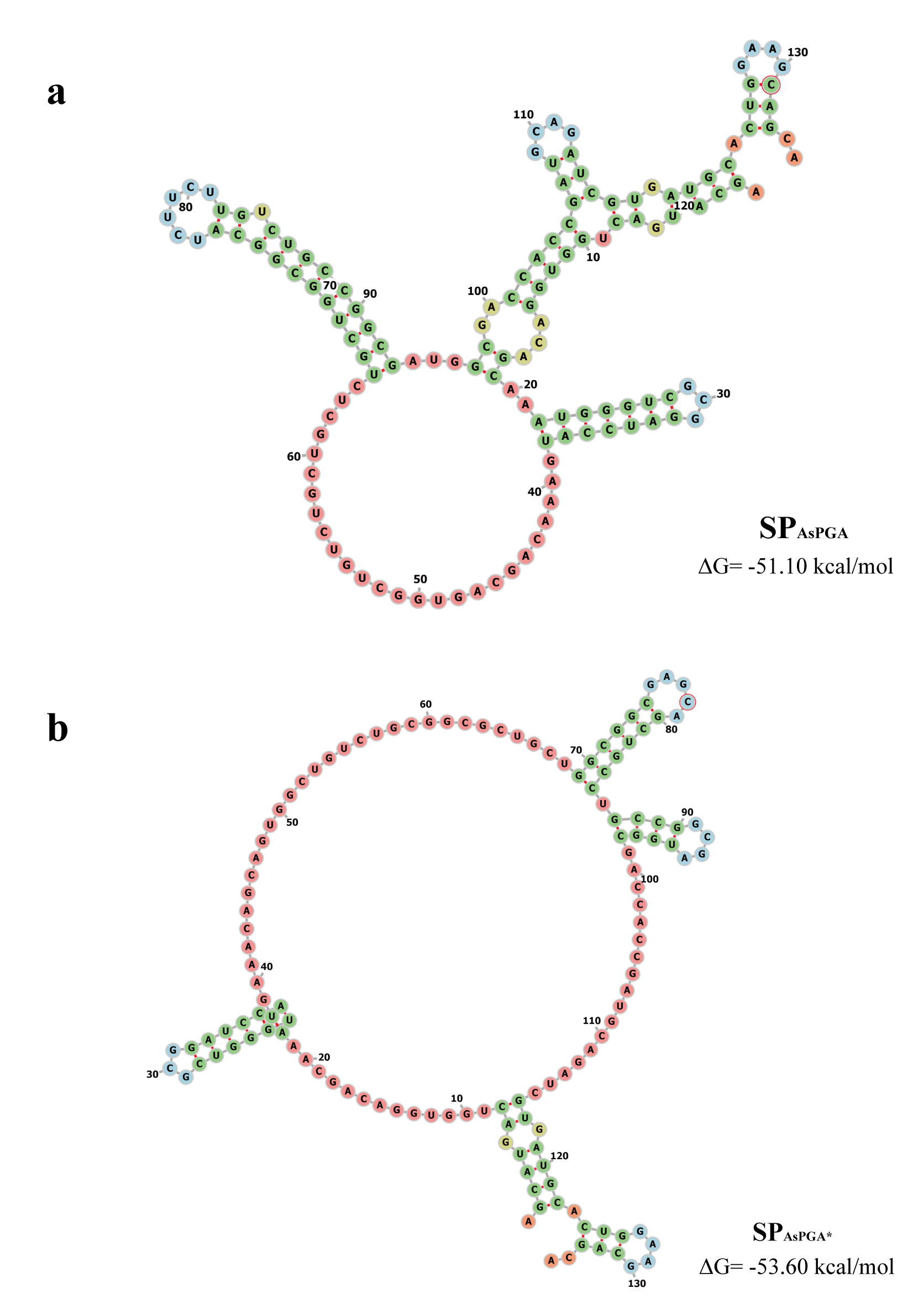


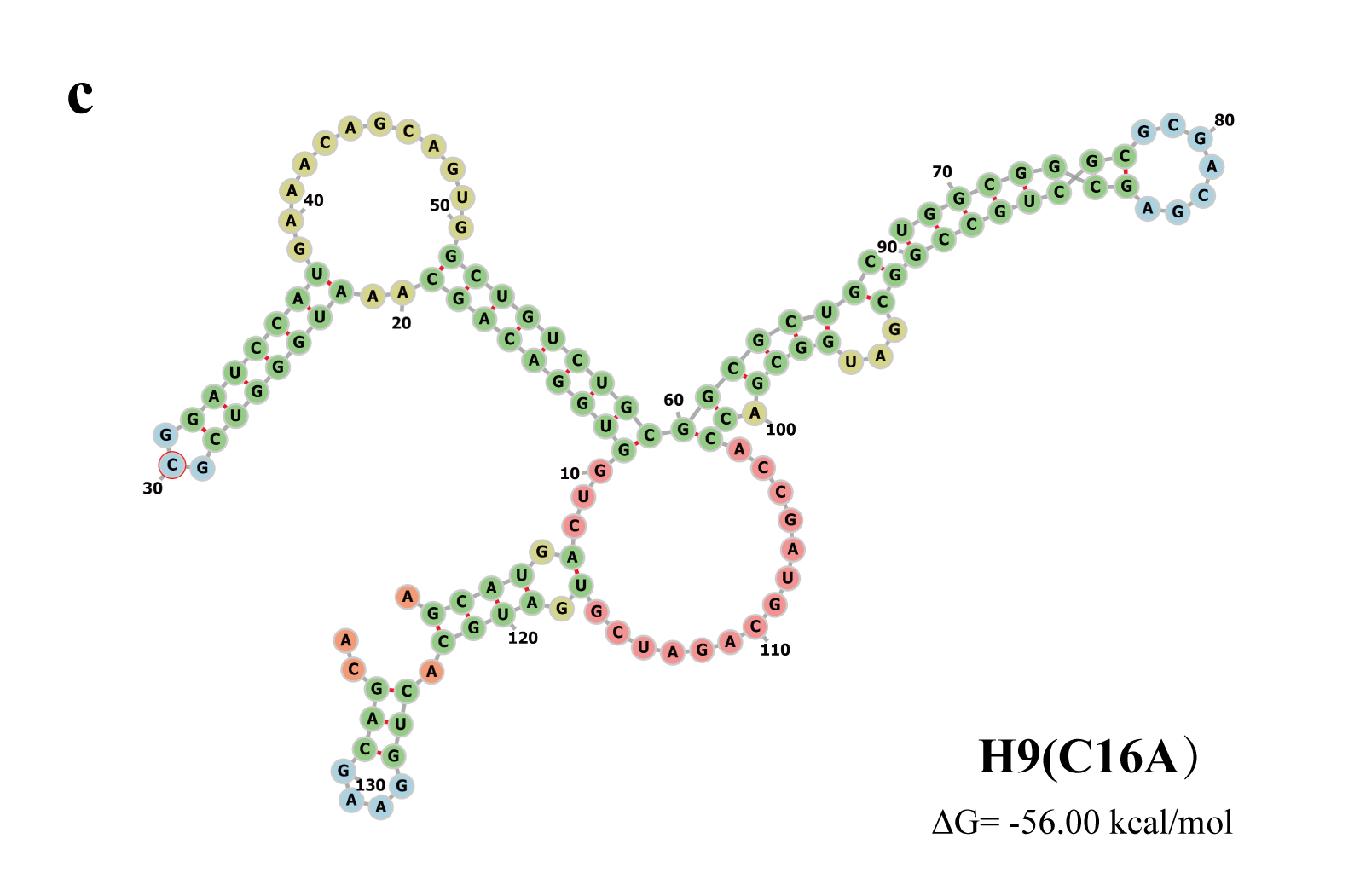


Fig. S9 Predicted mRNA secondary structures of the signal peptide coding region.The mRNA secondary structures corresponding to the signal peptide coding sequences were predicted using RNAfold (ViennaRNA package). The minimum free energy (MFE) values were -51.10 kcal/mol for SP_AsPGA_, -53.60 kcal/mol for SP_AsPGA*_, and -56.00 kcal/mol for H9(C16A).
